# Diminished Antioxidant Activity of High‐Density Lipoprotein–Associated Proteins in Chronic Kidney Disease

**DOI:** 10.1161/JAHA.112.000104

**Published:** 2013-04-24

**Authors:** David J. Kennedy, W. H. Wilson Tang, Yiying Fan, Yuping Wu, Shirley Mann, Michael Pepoy, Stanley L. Hazen

**Affiliations:** 1 Department of Cellular and Molecular Medicine, Cleveland Clinic, Cleveland, OH (D.J.K., W.T., S.L.H.); 2 Center for Cardiovascular Diagnostics and Prevention, Lerner Research Institute, Cleveland Clinic, Cleveland, OH (W.T., S.M., M.P., S.L.H.); 3 Department of Nephrology and Hypertension, Glickman Urological and Kidney Institute, Cleveland Clinic, Cleveland, OH (D.J.K.); 4 Department of Mathematics, Cleveland State University, Cleveland, OH (Y.F., Y.W.)

**Keywords:** arylesterase, chronic kidney disease, HDL, outcomes, oxidant stress, paraoxonase‐1

## Abstract

**Background:**

Decreased serum arylesterase activity, catalyzed by the high‐density lipoprotein–associated paraoxonase (PON)‐1, is associated with increased oxidant stress and atherosclerosis risk. We sought to determine the prognostic value of serum PON‐1 activity, as monitored by PON or arylesterase activities, in subjects with chronic kidney disease (CKD), particularly in relation to established cardiac biomarkers.

**Methods and Results:**

Serum arylesterase and PON activities were measured in sequential subjects with CKD (n=630; estimated glomerular filtration rate [eGFR] <60 mL/min per 1.73 m^2^) and an age‐ and sex‐matched control group of non‐CKD subjects (n=315) presenting for cardiac evaluations and prospectively followed for incident (3‐year) major adverse cardiac events (composite of death, nonfatal myocardial infarction, and stroke). Serum arylesterase activity in CKD subjects was lower compared with that in non‐CKD control subjects [median (interquartile range) 94 (77 to 112) versus 103 (85 to 121) μmol(L·min) per mL, *P*<0.001]; similarly, PON activity in CKD subjects was lower compared with that in non‐CKD control subjects [median (interquartile range) 474 (275 to 936) versus 586 (301 to 1118) nmol(L·min) per mL, *P*<0.001]. Lower serum arylesterase (hazard ratio 1.8, 95% CI 1.26 to 2.57, *P*<0.01) was a predictor of poorer outcomes. After adjusting for traditional risk factors and medication use, lower serum arylesterase (hazard ratio 1.55, 95% CI 1.08 to 2.23, *P*<0.05) still conferred an increased risk of major adverse cardiac events at 3 years.

**Conclusions:**

In patients with CKD, decreased serum arylesterase activity, a measure of diminished antioxidant properties of PON‐1, predicts higher risk of incident long‐term adverse cardiovascular events (heart attack, stroke, or death) in multivariable models adjusting for established clinical and biochemical risk factors.

## Introduction

Oxidative stress is an important participant in the pathogenesis and progression of chronic kidney disease (CKD).^1–2^ Stable byproducts of oxidative stress, including oxidized low‐density lipoproteins (LDL),^3–4^ malondialdehyde,^5^ isoprostanes,^6^ and carbonylated proteins,^7–8^ are increased in uremic conditions. In the setting of renal disease, increased oxidant generation coupled with depressed endogenous antioxidant mechanisms generates a redox imbalance that favors oxidative stress.^9^ This imbalance between prooxidant and antioxidant mechanisms may contribute to glomerular and tubulointerstitial damage and progression of CKD.^10^ Indeed, in the SPACE (Space‐Protected Angioplasty versus Carotid Endarterectomy) trial, a small double‐blind placebo‐controlled randomized trial in which alpha tocopherol (vitamin E) 800 IU, a presumed antioxidant therapy, was provided to subjects undergoing maintenance hemodialysis, significant reductions in cardiovascular events were observed.^11^ Although there is significant evidence that enhanced oxidant stress is observed in subjects with CKD and end‐stage renal disease (ESRD), the pathways responsible for this imbalance are still under investigation.

In addition to enhanced oxidant stress, subjects with CKD exhibit lipoprotein abnormalities such as increased remnant particles and triglycerides and both deficiency and dysfunction in high‐density lipoprotein (HDL), a presumed atheroprotective lipoprotein with mechanistic links to atherosclerosis and cardiovascular disease in both subjects with normal renal function and patients with ESRD.^12–15^ Paraoxonase (PON)‐1 is a glycoprotein that associates with HDL and is believed to facilitate some of its systemic antioxidant activities, including protection against lipoprotein oxidation and remodeling of oxidized phospholipids.^16–17^ Although its endogenous substrate(s) remains a topic of study, PON‐1 is mechanistically linked to multiple systemic indices of oxidant stress both in animal models of disease^18–19^ and within humans.^20^ PON‐1 activity within serum is functionally characterized by its hydrolysis organophosphate compounds such as paraoxon and carboxylic esters such as phenyl acetate. These PON and arylesterase activities have been linked both clinically and experimentally with multiple systemic measures of oxidant stress and have established the role of PON‐1 as an important antioxidant enzyme.^18,20^ Recent genomewide association studies of systemic PON and arylesterase activities in humans confirmed that genetic variants within the PON‐1 locus control systemic PON and aryleseterase activities.^21^ In addition to its antioxidative properties, PON‐1 has been mechanistically linked to protection from atherosclerosis risk,^18,20^ including promoting antiatherogenic properties against macrophage cholesterol accumulation, one of the earliest cellular hallmarks of the atherosclerotic process.^22–23^ In the current study, we examined the potential role of HDL antioxidant activity, as monitored by systemic measures of serum PON‐1 arylesterase and PON activities, as a predictor of cardiovascular disease progression and incident adverse events, myocardial infarction, stroke, and death, among patients with moderate CKD.

## Methods

### Study Population

We performed serum arylesterase activity and PON activity in serum samples collected from a prospective cohort of 630 consecutive individuals with CKD (estimated glomerular filtration rate [eGFR] <60 mL/min per 1.73 m^2^) who presented for elective diagnostic cardiac evaluations at a tertiary referral hospital. All subjects were stable and underwent elective diagnostic coronary angiography (either cardiac catheterization or coronary computed tomography angiography) not in the setting of acute coronary syndrome (cardiac troponin I <0.03 μg/L) with blood samples collected before any heparin administration. None of the study subjects were receiving dialysis. A control group of non‐CKD subjects (eGFR ≥60 mL/min per 1.73 m^2^, n=315) was obtained from the same setting and matched for age and sex (1:2 versus the CKD subjects). Serum samples in all subjects were collected in serum separator tubes, processed, and stored in aliquots at −80°C within 4 hours of phlebotomy. All participants gave written informed consent, and the Institutional Review Board of the Cleveland Clinic approved the study protocols.

Data were recorded for standard cardiac risk factors, including age, sex, history of diabetes mellitus, cigarette smoking, systolic blood pressure, and fasting lipids. Estimated GFR was calculated according to the Kidney Disease Outcomes Quality Initiative Modification of Diet in Renal Disease guidelines.^24^ Creatinine, fasting blood glucose, and lipid profiles were measured on the Abbott Architect platform (Abbott Laboratories).

### End Points

Major adverse cardiovascular events (MACEs) were defined as death, nonfatal myocardial infarction, or nonfatal cerebrovascular accident after enrollment. End points were collected by in‐person prospective follow‐up including letter solicitation and reply cards, chart review, and direct contact by study staff. We ascertained adjudicated outcomes over the ensuing 3 years for all participants after enrollment.

### Serum Biochemical Assays

Venous blood samples were collected in and immediately processed and frozen at −80°C until analysis. Serum arylesterase activity level was determined using a modification of a spectrophotometry‐based assay as previously described.^21^ Briefly, initial hydrolysis rates at 25°C of phenyl acetate substrate (3.6 mmol/L) were determined at 270 nm in 1:50 diluted serum in reaction mixtures consisting of Tris hydrochloride 9 mmol/L, pH 8.0, and calcium chloride 0.9 mmol/L in a 96‐well plate format (Spectramax 384 Plus; Molecular Devices). An extinction coefficient (at 270 nm) of 1310 mol/L per cm was used for calculating units of arylesterase activity in serum, which are expressed as the amount of phenyl acetate hydrolyzed in units of μmol(L·min) per mL. The intraassay and interassay coefficients of variance for arylesterase activity assay were each <4.0% when performed on 20 replicates performed on 10 different days over the course of assay. Serum PON activity was measured spectrophotometrrically in an open channel on the aforementioned Architect ci8200 platform (Abbott Laboratories). The *para‐*nitrophenol generation was determined at 405 nm in 1:40 diluted serum in reaction mixtures containing paraoxon 1.5 mmol/L (Sigma‐Aldrich, St Louis, Missouri), Tris hydrochloride 10 mmol/L, pH 8.0, sodium chloride 1 mol/L, and calcium chloride 2 mmol/L at 24°C. PON activity units were calculated using an extinction coefficient (at 405 nm) of 17 000 mol/L per cm, and values were expressed as nanomoles of *para*‐nitrophenol produced per minute per milliliter of serum. Intraassay and interassay coefficients of variance for the PON activity assay used were each <3.5% as determined from 30 replicates performed on 15 different days during the course of sample analyses. All other biochemical assays were performed on the Architect ci8200 platform according to the manufacturer's guidelines.

### Statistical Analysis

We compared baseline characteristics between subjects with high versus low serum arylesterase and PON activity levels by means of the Student *t* test (normally distributed) or Wilcoxon rank sum test (non–normally distributed) for continuous variables and χ^2^ test for categorical variables. The Spearman correlation was performed to determine the relationship between serum arylesterase and PON activity levels and other biochemical parameters. For each continuous variable, we investigated the log‐linearity assumption of Cox models by introducing a cubic spline component. Receiver operator characteristic curve analyses in the context of the time to event were performed to determine the optimal cutoff at <70 μmol(L·min) per mL (arylesterase) and <280 nmol(L·min) per mL (PON) activity levels, with risk of event estimated using 5‐fold cross‐validation by a Cox model. Both net reclassification improvement (NRI) and integrated discrimination improvement (IDI) were used to quantify improvement in model performance. The *P* values compare models with and without arylesterase and PON. Both models were adjusted for traditional risk factors, including age, sex, smoking, HDL cholesterol, systolic blood pressure, history of diabetes mellitus, triglyceride, and creatinine clearance. Cutoff values for NRI estimation used a ratio of 6:3:1 for low‐, medium‐, and high‐risk categories. Kaplan–Meier analysis with log‐rank test was used to compare the survival curves of the 2 groups of the respective PON‐1 activity levels [serum arylesterase activity <70 versus ≥70 μmol(L·min) per mL, serum PON activity <280 versus ≥280 nmol(L·min) per mL]. Cox proportional hazards regression was used for time‐to‐event analysis to determine hazard ratios (HRs) and 95% CIs for MACEs. The analysis was adjusted for individual traditional cardiac risk factors, including age, sex, systolic blood pressure, cigarette smoking, and fasting cholesterol values (including LDL and HDL cholesterol levels). Additional adjustments included medication use (including angiotensin‐converting enzyme inhibitors, angiotensin II receptor blockers, β‐blockers, and statin therapy), to predict incident 3‐year MACE risks. For non–log‐linear variables such as serum arylesterase activity, the continuous variable was transformed into a binary variable, and the optimal cutoff value for dichotomization was selected as that value that minimized the prediction error in MACEs. All analyses were performed using SAS version 8.2 (SAS Institute) and R 2.8.0 (www.r-project.org), and statistical significance was considered to be *P*<0.05.

## Results

### Subject Characteristics

**Figure 1.**
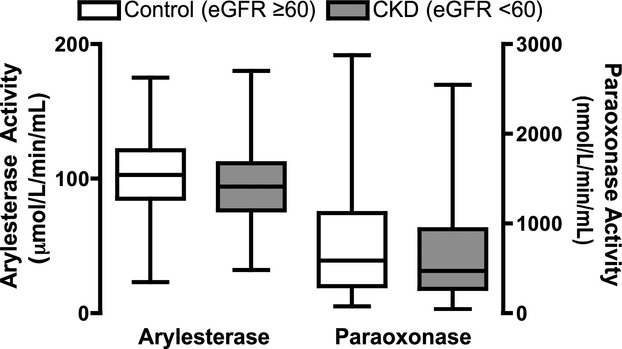
Comparison of serum arylesterase activity (left) and serum paraoxonase activity (right) between control subjects (eGFR ≥60 mL/min per 1.73 m^2^) and patients with chronic kidney disease (eGFR <60 mL/min per 1.73 m^2^). Probability value <0.001 versus control for arylesterase and paraoxonase by both Wilcoxon and *t* test. CKD indicates chronic kidney disease; eGFR, estimated glomerular filtration rate.

Baseline characteristics of the study population are given in Table 1. The mean and median eGFR were 46±13 and 50 mL/min per 1.73 m^2^ (interquartile range 40 to 56 mL/min per 1.73 m^2^) in the CKD subjects. Serum arylesterase activity in CKD subjects was lower compared with that in non‐CKD control subjects (median [interquartile range] 94 [77 to 112] versus 103 [85 to 121] μmol(L·min) per mL, *P*<0.001, Figure 1); similarly, PON activity in CKD subjects was lower compared with that in non‐CKD control subjects (median [interquartile range] 474 [275 to 936] versus 586 [301 to 1118] nmol(L·min) per mL, *P*<0.001, Figure 1). The baseline characteristics of the CKD population stratified by quartiles of serum arylesterase and PON activity are presented in Tables S1 and S2. Within the CKD subjects, serum arylesterase activity was inversely correlated with several measures of inflammation, including myeloperoxidase (*r*=−0.09, *P*<0.05) and C‐reactive protein (*r*=−0.12, *P*<0.05); however, these same trends were not significant for PON activity.

**Table 1.** Baseline Subject Characteristics

	CKD (n=630)	Control (n=315)	*P* Value
Age, y	69±10	70±10	0.79
Male, %	53	52	0.872
Diabetes, %	52	16	<0.001
Hypertension, %	86	61	<0.001
Cigarette smoking, %	61	57	0.292
LDL cholesterol, mg/dL	92 [73 to 115]	98 [82 to 114]	0.007
HDL cholesterol, mg/dL	32 [26 to 40]	39 [33 to 47]	<0.001
Triglycerides, mg/dL	134 [96 to 190]	91 [69 to 129]	<0.001
hsCRP, mg/L	4 [2 to 9]	2 [1 to 4]	<0.001
MPO, pmol/L	134 [89 to 288]	109 [72.1 to 298.7]	0.004
Serum arylesterase activity, μmol(L·min) per mL	94 [77 to 112]	103 [85 to 121]	<0.001
Serum paraoxonase activity, nmol(L·min) per mL	474 [275 to 936]	586 [302 to 1115]	0.006
Baseline medications, %			
ACE inhibitors/ARBs	63	37	<0.001
Beta‐blockers	66	42	<0.001
Statin	58	32	<0.001
Aspirin	66	55	<0.001

Values expressed as mean±SD or median [interquartile range]. CKD indicates chronic kidney disease; LDL, low‐density lipoprotein; HDL, high‐density lipoprotein; hsCRP, high sensitivity C reactive protein; MPO, myeloperoxidase; ACE, angiotensin‐converting enzyme; ARB, angiotensin II receptor blocker.

### Serum Arylesterase and PON Activity and Major Adverse Cardiac Outcomes

**Figure 2.**
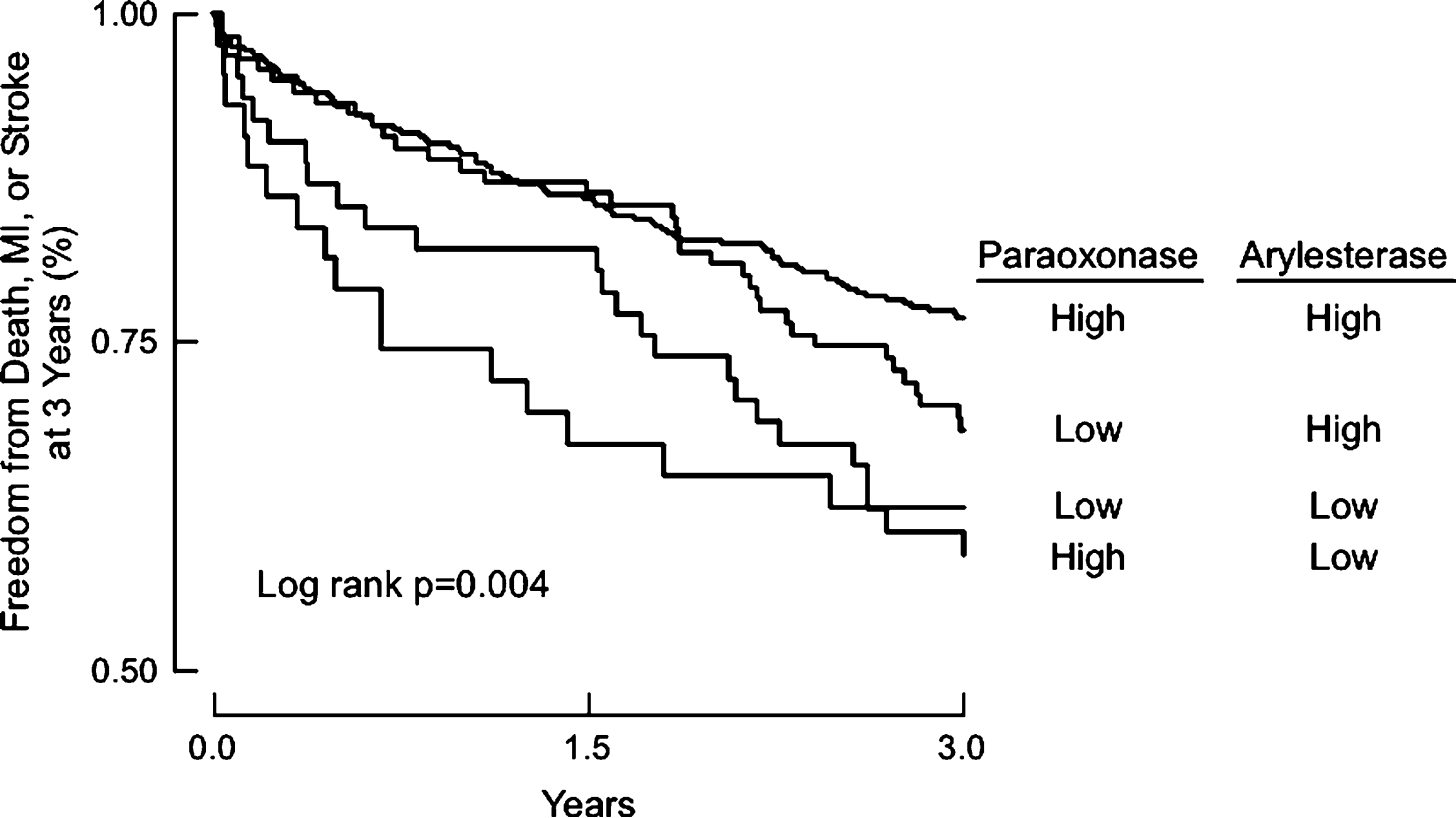
Kaplan–Meier analysis of major adverse cardiac events in patients with chronic kidney disease. Patients stratified according to optimal cutoff for serum arylesterase and paraoxonase activity levels as follows: “high” paraoxonase [≥280 nmol(L·min) per mL] or “low” paraoxonase [<280 nmol(L·min) per mL] and “high” arylesterase [≥70 μmol(L·min) per mL] or “low” arylesterase [<70 μmol(L·min) per mL].MI indicates myocardial infarction.

A total of 166 events (nonfatal myocardial infarction, stroke, or death) were recorded within the 3‐year period of follow‐up. When divided as a dichotomous variable according to optimal cutoff, lower serum arylesterase activity [<70 μmol(L·min) per mL] was predictive of future development of adverse cardiac events (hazard ratio 1.8, 95% CI 1.26 to 2.57, *P*<0.01; Table 2). Lower serum PON activity showed similar trends when divided by optimal cutoff [<280 nmol(L·min) per mL] but did not reach statistical significance (hazard ratio 1.35, 95% CI 0.98 to 1.88, *P*=NS; Table 2). After adjusting for traditional risk factors such as HDL and medication use including angiotensin‐converting enzyme inhibitors, angiotensin II receptor blockers, β‐blockers, and statin therapy, lower serum arylesterase still conferred an increased risk of major adverse cardiac events at 3 years (hazard ratio 1.55, 95% CI 1.08 to 2.23, *P*<0.05), whereas PON activity displayed similar trends but again failed to reach statistical significance (hazard ratio 1.22, 95% CI 0.87 to 1.71, *P*=NS). When the risk analysis was performed according to quartiles of either arylesterase or PON activity, the lowest serum arylesterase quartile (<77 μmol(L·min) per mL) was predictive of future development of adverse cardiac events (hazard ratio 2.17, 95% CI 1.4 to 3.37, *P*<0.001; Table S3). Subjects in the lowest serum PON activity quartile (<275 nmol(L·min) per mL) showed similar trends (hazard ratio 1.73, 95% CI 1.11 to 2.7, *P*<0.05; Table S3). After adjustment for traditional risk factors and medication use, the lowest serum arylesterase quartile still conferred an increased risk of major adverse cardiac events at 3 years (hazard ratio 1.64, 95% CI 1.03 to 2.60, *P*<0.05), whereas PON activity displayed similar trends but failed to reach statistical significance (hazard ratio 1.54, 95% CI 0.97 to 2.44, *P*=0.064). These findings were essentially the same when PON activity was adjusted for in the arylesterase model or when arylesterase was adjusted for in the PON model (Table S4). Moreover, the addition of serum PON or arylesterase activity to traditional risk factors resulted in significant integrated discrimination improvement (PON IDI 4%, *P*<0.001; arylesterase IDI 4%, *P*<0.001) and significant event‐specific net reclassification (PON NRI 12%, *P=*0.01; arylesterase NRI 10%, *P=*0.02). Kaplan–Meier survival analyses demonstrated that the combination of low serum arylesterase and PON activity levels within CKD subjects were associated with higher event rates compared with those with higher activity levels of arylesterase or PON (log rank *P*=0.004; Figure 2).

**Table 2.** Unadjusted and Adjusted 3‐Year Hazard Ratio for MACE at 3 Years Stratified by Optimal Cut‐Off Values for Serum Arylesterase and Paraoxonase Activity Levels

	Arylesterase activity, μmol(L·min) per mL (range)		Paraoxonase activity, nmol(L·min) per mL (range)	
Range	<70	≥70	<280	≥280
3‐y MACE, %	41/107	125/523	52/161	114/469
Unadjusted HR	1.80 (1.26 to 2.57)**	1	1.35 (0.98 to 1.88)	1
Adjusted HR	1.55 (1.08 to 2.23)*	1	1.22 (0.87 to 1.71)	1

Model adjusted for traditional risk factors including age, sex, systolic blood pressure, low‐density lipoprotein cholesterol, high‐density lipoprotein cholesterol, smoking, diabetics, and ACE inhibitor, ARB, β‐blocker, and statin use. MACE indicates major adverse cardiac events; HR, hazard ratio; ACE, angiotensin‐converting enzyme; ARB, angiotensin II receptor blocker. *P<0.05, **P<0.01.

## Discussion

In the current study, we provide evidence of a significant reduction in HDL‐associated antioxidant PON‐1 activity (as quantified by both serum arylesterase and PON activity) in a large cohort of stable mild–moderate CKD patients undergoing elective coronary angiography, compared with age‐ and sex‐matched control subjects without CKD. Furthermore, we demonstrate a significant association between both low serum arylesterase and PON activities and poor long‐term prognosis independent of traditional cardiac risk factors. To our knowledge, this is the largest study to examine the relationship between arylesterase and PON activities and clinical outcomes in subjects with CKD. Although the HDL‐associated protein PON‐1 has established links with multiple measures of systemic oxidant stress and atherosclerotic risk in subjects with normal renal function,^20–21^ the current findings imply a potential importance for PON‐1 as an important protective pathway that is diminished in the setting of even moderately reduced renal function. Thus, PON‐1 may be a clinically useful diagnostic and therapeutic target in the setting of CKD because these patients have limited treatment options that address their significant burden of cardiovascular morbidity and mortality.

Although there is a paucity of knowledge regarding the role of PON‐1 across the spectrum of CKD, its role in chronic renal failure and ESRD has recently been reviewed.^25^ Indeed, PON activity and expression is lower in patients with ESRD,^26–27^ a finding that has been repeated in multiple ESRD cohorts.^28–30^ Not only does hemodialysis increase the antiinflammatory activities of HDL,^31^ but also increases in PON‐1 activity are observed after long‐term hemodialysis,^32^ suggesting that uremic toxins may play a mechanistic role in the suppression of PON‐1 activity. Interestingly, toxins such as the reactive aldehyde acrolein are elevated in uremia, removed via hemodialysis, and demonstrate dose‐ and time‐dependent PON inactivation that is attenuated with *N*‐acetylcysteine.^33^ Similar to hemodialysis, renal transplantation also appears to normalize PON activity in ESRD.^27^ Interestingly, changes in PON‐1 activity within the chronic renal failure population do not appear to be linked to genetic polymorphisms,^34–35^ whereas genetic polymorphisms are linked to systemic PON activity measures in subjects with normal renal function.^20,36–37^

The precise pathophysiologic mechanism whereby decreased PON‐1 activity leads to major adverse cardiac events in CKD is not fully understood. In addition to its esterase activity, PON‐1 is both a lactonase and a thiolactonase.^38^ There is some evidence to suggest that the increased lipid peroxidation and protein homocysteinylation detected in ESRD patients are associated with diminished lactonase activity and that hemodialysis is capable of restoring lactonase activity to levels indistinguishable from those of control subjects.^39^ In a small study (N=60) of subjects with ESRD, diminished PON‐1 activity was correlated with carotid intimal‐medial thickness measures,^40^ and elevated homocysteine–thiolactone levels found in ESRD have also been correlated with both diminished PON‐1 activity and decreased antiatherogenic capacity.^39,41–42^ In the present study, we noted that serum arylesterase activity was inversely correlated with serum levels of both myeloperoxidase and CRP, suggesting that the ability of PON to modulate inflammatory processes in CKD may be responsible in part for the observed benefit in terms of cardiovascular risk.

The current study extends our findings of the prognostic value of serum PON and arylesterase activities to a large population of patients with mild‐to‐moderate CKD with prospective long‐term clinical outcomes. We previously reported that both PON‐1 polymorphisms and activity have significant independent and additional prognostic value beyond standard clinical, biochemical, and echocardiographic parameters in stable patients undergoing diagnostic coronary angiography.^20^ Interestingly, the presence of subclinical myocardial necrosis in these patients is also associated with reduction in arylesterase activity.^43^ Our results are consistent with another population with end‐organ dysfunction—patients with systolic heart failure, in whom decreased serum arylesterase activity predicts higher risk of incident long‐term adverse cardiac event independent of established clinical and biochemical risk factors.^44^ The present finding raises the possibility that a specific antioxidative pathway, such as catalyzed by PON‐1, modulates cardiovascular risk even at the earlier stages of CKD and warrants further mechanistic investigations.

### Study Limitations

>In addition to the selection bias that accompanies all case–control studies, serum arylesterase and PON activities were only measured at a single time point. We are thus limited in our ability to determine the impact of therapeutic effects on PON‐1 activity levels over time or whether changes in PON‐1 activity levels over time yield any further prognostic value in patients with CKD. Furthermore, the fact that PON activity was not predictive of long‐term adverse cardiac events in the Cox proportional hazards model may be reflective of either an insufficient sample size or the fact that the promiscuous esterase activity of PON‐1 is not entirely reflective of changes in its physiologic function. To be sure, there is some agreement that the main physiologic activity of PON‐1 is its lactonase activity,^41^ which may in fact rely on a different active site of PON‐1 than that of its esterase activity.^45^ Because we did not perform measurement of lactonase activity in these samples, we cannot comment on whether the strong association of lower PON‐1 lactonase activity seen in a small‐cohort study in an ESRD population^39^ also holds true in these patients with moderate CKD. We do note, however, that the present study, in contrast to prior cross‐sectional studies, shows low arylesterase activity heralds increased prospective risk for MACEs over the ensuing 3‐year period. A further limitation is that we do not have complete PON‐1 polymorphism data for these patients. We thus cannot adequately assess the contribution of genetic factors in this population, although the prevalence of 192 Gln/Arg and 55 Leu/Met PON‐1 polymorphisms reportedly do not appear to differ between patients with chronic renal failure and control subjects.^34^ Despite these limitations, to our knowledge, this study represents the first examination of the contribution of PON‐1 activity to long‐term adverse cardiac events in a large cohort of well‐characterized subjects with moderate CKD. Because patients with CKD are disproportionately burdened by both oxidant stress and cardiovascular disease, these findings warrant further examination of this important antioxidative pathway.

## Conclusion

In patients with moderate CKD, diminished activity levels of the antioxidant HDL‐associated enzyme PON‐1, as monitored by serum arylesterase and PON activities, predict increased risk for the development of adverse cardiac events, including nonfatal myocardial infarction, nonfatal stroke, or death, in multivariable models adjusted for established clinical and biochemical risk factors. These findings further support the hypothesis that oxidative stress is an important mediator in the progression of kidney disease and point to the potential antioxidant compensatory role of HDL and its associated protein PON‐1. Studies aimed at modulating PON‐1 activity for both cardioprotective effects, and also possibly even as a means of potentially protecting the kidney from disease progression, merit consideration in this population of patients with substantial cardiovascular morbidity and mortality.
